# Higher risk of renal impairment associated with tenofovir use amongst people living with HIV in India: A comparative cohort analysis between Western India and United Kingdom

**DOI:** 10.1186/1471-2334-14-173

**Published:** 2014-03-29

**Authors:** Sanjay N Pujari, Colette Smith, Abhimanyu Makane, Mike Youle, Margaret Johnson, Vivek Bele, Kedar Joshi, Digamber Dabhade, Sanjay Bhagani

**Affiliations:** 1 Institute of Infectious Diseases, Pune, India; 2 University College London, London, UCL, UK; 3 Royal Free Hospital, London, UK

**Keywords:** Tenofovir, Nephrotoxicity, India

## Abstract

**Background:**

Data on the renal safety of Tenofovir (TDF) in Low and Middle Income Countries (LMICs) is scarce. We compared development of various forms of renal impairment with use of TDF-containing antiretroviral therapy (ART) between a cohort from the Institute of Infectious Diseases (IID) Pune, Western India and the Royal Free Hospital (RFH) London, UK.

**Methods:**

This is a retrospective analysis of change in estimated glomerular filtration rates (eGFRs) at 6, 12 and 24 months post TDF initiation using the Modification of Diet in Renal Disease (MDRD) equation. In people living with Human Immunodeficiency virus (PLHIV) with pre-TDF eGFR > 90 ml/min/1.73 m^2^ time to development of and factors associated with progression to eGFR < 60 ml/min/1.73 m^2^ were calculated using standard survival methods.

**Results:**

A total of 574 (59% Caucasian) at the RFH, and 708 (100% Indian ethnicity) PLHIV from IID were included. Baseline median eGFR were similar; RFH 102 (IQR 89, 117), IID 100 (82, 119). At 24 months, mean (SD) decline in eGFR was -7(21) at RFH (p < 0.0001) and -7(40) at IID (p = 0.001). Amongst those with pre-TDF eGFR > 90 ml/min/1.73 m^2^ PLHIV at IID were more likely to develop an eGFR < 60 ml/min/1.73 m^2^ (aHR = 7.6 [95% CI 3.4, 17.4] p < 0.0001) and had a faster rate of progression estimated using Kaplan Meier methods. Risk factors included age (per 10 years older: aHR = 2.21 [1.6, 3.0] p < 0.0001) and receiving concomitant ritonavir boosted Protease Inhibitor (PI/r) (aHR = 2.4 [1.2, 4.8] p = 0.01).

**Conclusions:**

There is higher frequency of treatment limiting renal impairment events amongst PLHIV receiving TDF in Western India. As TDF scale up progresses, programs need to develop capacity for monitoring and treatment of renal impairment associated with TDF.

## Background

First line antiretroviral treatment (ART) regimens in Low and Middle Income Countries (LMICs) of non nucleoside reverse trancriptase inhibitor (NNRTI) with thymidine analogue nucleoside reverse transcriptase inhibitors (tNRTIs) are associated with numerous long term toxicities [1]. The World Health Organization (WHO) has recommended phasing out stavudine (d4T) and using tenofovir (TDF) as component of first line treatment regimens [2]. Amongst those failing first-line tNRTI regimens, TDF with ritonavir boosted PI (PI/r) is recommended as a second line regimen.

Tenofovir based regimens are associated with excellent efficacy and safety in clinical trials [3]. However, these studies have been done in High Income Countries (HICs) and data on their effectiveness and safety in LMICs is limited [4-7]. Tenofovir has been associated with renal toxicity amongst 1-2% of people living with Human Immunodeficiency Virus (PLHIV) but higher proportion of patients demonstrate a fall in estimated Glomerular Filtration Rate (eGFR) [8]. Traditional risk factors are common amongst PLHIV experiencing renal toxicity to antiretrovirals, although not everybody with these risk factors develops renal adverse events [9]. In Sub-saharan Africa, severe eGFR impairment was infrequent over 4 years irrespective of the use of TDF [10]. The authors of this study conclude that TDF based first line ART can be safely given even without renal monitoring in these settings.

There is no published data on the incidence of TDF induced renal dysfunction amongst PLHIV in India. We determined and compared the incidence of and risk factors for renal impairment associated with TDF use amongst PLHIVs accessing care in a HIC and LMIC setting.

## Methods

### Setting

People living with HIV accessing care at two clinics: HIC-the Royal Free Hospital (RFH), London, UK and LMIC- Institute of Infectious Diseases (IID), Pune Western India were included

### Design

This is a retrospective study comparing renal outcomes amongst PLHIV at these two sites. Data was abstracted from clinic electronic and paper records. Approval for the study was given by Chairman’s approval letter from the Royal Free Ethics Committee while at Institute of Infectious Diseases all HIV positive persons provide consent on Ethics committee (Chest Research Foundations Independent Ethics Committee, Pune India) approved form mentioning that data collected during the course of care would be used for research analysis. Patient details were annonymized for data analysis.

### Study population

All HIV-1 positive adults initiating TDF for the first time between 1^st^ January 2007 to 31^st^ December 2009 with at least one follow up eGFR measurement were included in the final analysis. At both sites renal evaluations (serum creatinine, urine analysis, serum phosphate, electrolytes) are performed at baseline and every 3 to 6 months after initiation of TDF as standard of care and whenever clinically indicated. At IID PLHIV pay out of pocket for subsidized antiretrovirals (ARVs) and laboratory tests, while National Health Services (NHS) provides for the same at RFH.

### Measurements

The eGFR was estimated using the Modification of Diet in Renal Disease (MDRD) equation. However, all analyses were repeated using the Cockroft-Gault (CG) formula and the results were consistent. A recent study amongst Indians has documented optimal performance of MDRD equation for estimating eGFR, while the Chronic Kidney Disease Epidemiology Collaboration (CKD-EPI) equation showed marked bias at higher eGFRs [11]. Information on demographic variables at the time of initiating TDF was summarised. An eGFR >90/ml/min/1.73 m^2^ is considered normal while eGFR <60 ml/min/1.73 m^2^ considered as decreased. Partial Fanconi’s syndrome was diagnosed by the presence of one or more of the following: normoglycemic glycosuria, hypophosphatemia, tubular acidosis and hypouricemia [12]. CD4 counts and Plasma viral loads were determined by flow cytometry and PCR (Roche Taqman, Lower limit of quantification <40 copies/ml) respectively. Treatment limiting renal toxicity was defined as any renal impairment leading to permanent discontinuation of TDF, including those who demonstrated a declining trend to an eGFR of less than 60 ml/min/1.73 m^2^.

### Data analysis

Characteristics of PLHIV at the time of starting TDF were summarised according to clinical centre. The eGFR at the time of initiating TDF (Baseline; measured within the 6 months previous to initiating TDF), and 6, 12, 18 and 24 months after starting TDF were calculated by creating a window 3 months either side of the time point of interest, and considering the eGFR that occurred within the window closest to this time point. As the eGFR measurements were suitable normally distributed, the eGFR value at each time point, as well as the change in eGFR from baseline were summarised using the mean and standard deviation (SD). Changes over time within and between centres were compared using paired and unpaired t-tests respectively at two pre-defined time points of 6 and 24 months post-TDF initiation.

Finally, the subset of PLHIV with an eGFR >90 ml/min/1.73 m^2^ when initiating TDF were considered. Time to development of eGFR < 60 ml/min/1.73 m^2^ was calculated using standard survival methods, with individuals who did not experience the event being censored at the time of the last available eGFR measurement. Factors associated with time to eGFR < 60 ml/min/1.73 m^2^ were investigated using Cox proportional hazards models. The variables included in this models included site, age, gender, use of concomitant PI/r, pre-TDF and nadir CD4 count, time since HIV diagnosis, and ever receiving PI/r prior to receipt of TDF. No formal adjustments were made for multiple testing. All analyses were performed using SAS version 9.3 (SAS Institute Inc, Cary, NC).

## Results

### Baseline demographics

A total of 574 and 708 HIV positive individuals from RFH and IID respectively initiating TDF based ART were included in the final analysis (Table 1). All PLHIV at IID were of Indian ethnicity whilst approximately 60% at RFH were Caucasian. Approximately 75% PLHIV at IID were using TDF in an NNRTI based (EFV, NVP) regimen whilst 56% at RFH used TDF in a ritonavir-boosted PI-based regimen. Approximately half of the PLHIV at each site were ARV naive at TDF initiation. At both the sites EFV was the commonest NNRTI used (36.8% at RFH and 48.2% at IID) while LPV/r (26.5%) and ATV/r (17.5%) were the main PIs used at RFH and IID respectively. Median CD4 count at baseline was lower at IID (242 cells/mm^3^ vs 348 cells/mm^3^). Measurement of viral load prior to initiation of ART is not part of routine care at IID. Baseline median eGFR were normal and similar at both sites (102 ml/min/1.73 m^2^ and 100 ml/min/1.73 m^2^ at RFH and IID respectively). Approximately one-third of PLHIV at both sites had eGFR < 90 ml/min/1.73 m^2^ at baseline (Table 2).

**Table 1:** Patient characteristics at time of starting tenofovir

		**Royal Free**	**Pune**
N		574 (100.0)	708 (100.0)
Age (years)	Median (IQR)	41 (35, 47)	38 (34, 45)
Gender	Male	439 (76.5)	537 (75.8)
	Female	135 (23.5)	171 (24.2)
Ethnicity	White	339 (59.1)	-
	Black	161 (28.1)	-
	Indian	-	708 (100.0)
	Other	74 (12.9)	-
Risk for HIV transmission	MSM	333 (58.0)	-
	Heterosexual	224 (39.0)	-
	Other	17 (3.0)	-
CD4 count (cells/mm^3^)	Median (IQR)	348 (230, 525) N = 571	242 (118, 395) N = 657
HIV RNA viral load (log copies/ml)	Median (IQR)	4.2 (<1.7, 5.0) N = 536	N/A
ART-naïve when started TDF	Yes	314 (54.7)	307 (43.4)
	No	260 (45.3)	401 (56.6)
Time since first ART at time of starting TDF (weeks)	Median (IQR)	368 (217, 545) N = 260	162 (66, 274) N = 400
Other ARVs in regimen	Emtricitabine	530 (97.2)	641 (90.5)
	Lamivudine	28 (4.9)	61 (8.6)
	Zidovudine	10 (1.7)	62 (8.8)
	Didanosine	1 (0.2)	1 (0.1)
	Abacavir	22 (3.8)	2 (0.2)
	Lopinavir	152 (26.5)	49 (6.9)
	Atazanavir	88 (15.3)	125 (17.7)
	Darunavir	35 (6.1)	0 (0.0)
	Saquinavir	28 (4.9)	1 (0.1)
	Fos-amprenavir	16 (2.8)	0 (0.0)
	Nelfinavir	2 (0.4)	0 (0.0)
	Ritonavir	308 (53.6)	181 (25.6)
	Efavirenz	211 (36.8)	341 (48.2)
	Nevirapine	50 (8.7)	183 (25.9)
	Etravirine	7 (1.2)	0 (0.0)
	T20	1 (0.2)	0 (0.0)
	Raltegravir	13 (2.3)	0 (0.0)
	Maraviroc	2 (0.4)	0 (0.0)
	Stavudine		13 (1.8)
	Indinavir		6 (0.9)
Baseline eGFR	Median (IQR)	102 (89, 117)	100 (82, 119)
Baseline creatinine	Median (IQR)	77 (68, 87)	79 (62, 88)

**Table 2:** **GFR (ml/min/1.73 m**^**2**^**) results using MDRD over follow up**

	**Baseline**	**6 mths**	**12 mths**	**18 mths**	**24 mths**
**Royal Free patients**					
Number with measure	574	514	468	364	273
GFR Mean (SD)	104 (25)	100 (23)	99 (22)	97 (21)	97 (25)
Change from baseline	-	-4 (19)	-6 (18)	-6 (19)	-7 (21)
GFR <90	150 (26.1)	169 (32.9)	178 (38.0)	128 (35.2)	110 (40.3)
GFR < 60	10 (1.7)	7 (1.4)	8 (1.7)	9 (2.5)	11 (4.0)
GFR < 30	1 (0.2)	1 (0.2)	2 (0.4)	2 (0.6)	0 (0.0)
P-value for change from baseline		<0.0001			<0.0001
**IID, Pune patients**					
Number with measure	702	439	391	366	302
GFR Mean (SD)	105 (35)	97 (28)	96 (28)	98 (29)	96 (31)
Change from baseline		-6 (39)	-7 (38)	-8 (40)	-7 (40)
GFR <90	254 (36.2)	193 (44.0)	195 (49.9)	165 (45.1)	144 (47.7)
GFR < 60	38 (5.4)	28 (6.4)	27 (6.9)	32 (8.7)	23 (7.6)
GFR < 30	2 (0.3)	0 (0.0)	2 (0.5)	2 (0.6)	2 (0.7)
P-value for change from baseline		<0.0001			0.001
P-value for comparison of centres		0.46			0.76

### Changes in renal function

Changes in eGFR over follow up at the two sites are summarized in Table 2. There was a statistically significant decline in eGFR from baseline at 6 and 24 months at both the sites, however there was not a statistically significant difference in eGFR decline between sites. People living with HIV at both sites had an average decline in eGFR of 7 ml/min/1.73 m^2^ at 24 months post TDF-initiation.

**Figure 1:**
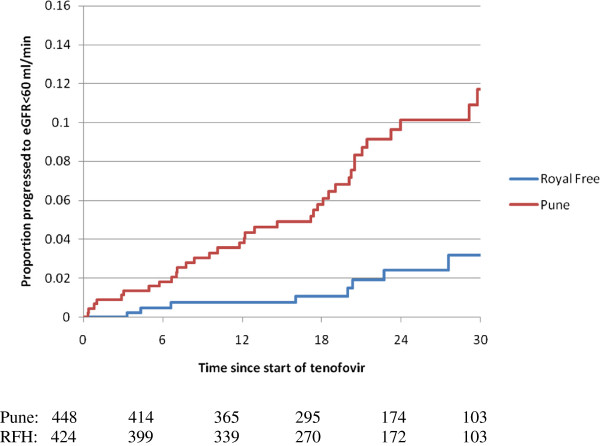
**Time to progression to eGFR < 60 m/min/1.73 m**^**2 **^**amongst those with baseline eGFR > 90 ml/min/1.73 m**^**2**^**.** Legend: Time to development of eGFR < 60 ml/min/1.73 m^2^ as determined by MDRD amongst HIV positive individuals initiating Tenofovir with a baseline eGFR > 90 ml/min/1.73 m2 at IID, Pune (red) and Royal Free Hospital, UK (blue). The difference across the centres is statistically significant (p < 0.0001).

Amongst individuals with pre-TDF eGFR > 90 ml/min/1.73 m^2^, PLHIV at IID were more likely than the RFH to develop an eGFR < 60 ml/min/1.73 m^2^ (adjusted hazard ratio [aHR]: 7.6 p < 0.0001) (Table 3 and Figure 1). Independent risk factors associated with progression to eGFR < 60 ml/min/1.73 m^2^ included age (per 10 years older aHR: 2.21) and receiving concomitant PI/r (aHR: 2.4). Interestingly, neither CD4 counts at eGFR estimation nor CD4 nadir were associated with significant eGFR decline.

**Table 3:** **Factors associated with HIV positive persons developing (single measurement) eGFR of <60 ml/min/1.73 m**^**2 **^**who had baseline eGFR > 90 /ml/min1.73/m**^**2**^

		**Univariate**			**Multivariate**		
		**HR**	**95% CI**	**p**	**HR**	**95% CI**	**p**
Centre	Pune	4.87	2.28, 10.38	<0.0001	7.65	3.36, 17.41	<.0001
	RF	1.00	-		1.00	-	
Age	Per 10 years older	1.85	1.43, 2.40	<.00001	2.21	1.61, 3.05	<.0001
Gender	Female	1.63	0.89, 2.99	0.12	1.72	0.90, 3.29	0.10
	Male	1.00	-		1.00	-	
Receiving PI	Yes	1.01	0.57, 1.79	0.97	2.40	1.21, 4.76	0.01
	No	1.00	-		1.00	-	
CD4 count	Per 100 cells	1.01	0.90, 1.13	0.89	1.04	0.89, 1.20	0.65
Time since diagnosis	Per year longer	0.98	0.92, 1.04	0.46	1.00	0.92, 1.07	0.92
CD4 nadir	Per 100 cells	0.94	0.78, 1.14	0.55	0.99	0.79, 1.23	0.91
Ever received a PI before TDF	Yes	0.76	0.32, 1.78	0.52	0.77	0.28, 2.15	0.62
	No	1.00	-		1.00	-	

An eGFR < 60 ml/min/1.73 m^2^ developed amongst 41 PLHIV at IID and 8 individuals at RFH. Fifteen PLHIV at IID developed partial Fanconi’s syndrome (8 with concomitant NNRTI and 7 with boosted PI). Fifty-nine (8.3%) individuals developed treatment limiting toxicity at IID (41with eGFR < 60 ml/min/1.73 m^2^, 15 partial Fanconi syndrome and 3 acute kidney injury) and 8 individuals developed treatment limiting toxicity (all with eGFR < 60 ml/min/1.73 m^2^) at RFH. Twenty-one (35.5%) of these PLHIV had co-morbidities (Hypertension-11, Diabetes -9, chronic HCV-1), however we did not perform any formal adjustment analysis due to small sample size. The characteristics of 15 PLHIV with partial Fanconi’s syndrome have been summarized in Table 4.

**Table 4:** Characteristics of HIV positive individuals with partial Fanconi’s syndrome

**Variable**	
Lab features	
Normoglycemic glycosuria with hypophosphatemia	11
Normoglycemic glycosuria with hypokalemia and metabolic acidosis	1
Normoglycemic glycosuria and hypouricemia	1
Hypophosphatemia	2
Medain age (range)	54.3 (42-67)
Gender	
Male	9
Median duration of TDF, weeks (range)	92 (24-188)
Co-morbidities	
None	13
Hypertension	2
Concomitant ARV	
NVP	5
EFV	3
ATV/r	5
LPV/r	2

## Discussion

To our knowledge, this is the first comparison of TDF associated renal dysfunction, amongst PLHIV accessing care in HIC and LMIC clinics. The WHO has recommended scaling up TDF based first line ARV regimens in LMICs. While we and others have demonstrated the effectiveness of TDF based first line regimens there is limited data on the safety, especially renal and bone toxicity in LMICs [13,14].

Although the change in eGFR from baseline were similar amongst PHIV at both sites, we found significantly higher frequency of treatment limiting renal toxicity and a higher risk of developing an eGFR < 60 ml/min/1.73 m^2^ associated with TDF in Western India. Weight was not included as a covariate in analysis, as when the results were repeated using the CG formula (which includes weight), consistent results were obtained However, weight/creatinine ratio has been associated with renal toxicity and perhaps current recommended dose of TDF may be high for Indians [15,16].

At both sites significant risk factors associated with reduction in eGFR were age and concomitant PI/r. Use of concomitant PI/r has been associated with higher risk of renal toxicity in individuals taking TDF [17]. Inhibition of Multi-Resistance Protein (MRP-4) efflux channels in the proximal convoluted tubules by ritonavir may contribute to this [18]. Renal dysfunction has been documented to be more common when TDF is combined with ATV/r as compared to LPV/r, although varying follow up times may have accounted for the difference in this study [19]. When TDF is used with PI/r in second line regimens in RLS, monitoring for renal toxicity is warranted. This also argues for using TDF in first line regimens with NNRTIs.

Risk factors associated with treatment limiting nephrotoxicity in our study included Diabetes, Hypertension, and receipt of concomitant nephrotoxic medications. It would be prudent to perform frequent renal monitoring or to avoid the use of TDF in the presence of these risk factors. Predictors of significant nephrotoxicity such as pre-existing renal impairment, older age, advanced HIV disease, vasculometabolic disease, low body weight have been well documented [16,20]. In addition careful renal monitoring is warranted amongst HIV positive individuals with acute inter-current illnesses.

The clinical presentations of treatment limiting renal toxicity seen at IID included progression to eGFR < 60 ml/min/1.73 m^2^, Acute Kidney Injury and partial Fanconi’s syndrome. Two of the HIV positive individuals were diagnosed as having partial Fanconi’s syndrome on the basis of only profound hypophosphatemia (serum phopshorus <1.5 mg/dl) and one patient with hypouricemia (Serum uric acid 1.9 mg/dl) and normoglycemic glycosuria. Although this is controversial, we did not have access to other tests for assessing tubular dysfunction like aminoaciduria, tubular proteinuria, and fractional excretion of phosphate. Partial Fanconi’s syndrome may present without eGFR decline, hence high clinical suspicion (e.g. proximal muscle weakness) and routine monitoring for glycosuria (triggering blood glucose determination to confirm normoglycemic glycosuria) is needed. Other components like aminoaciduria, tubular proteinuria and tubular acidosis needed to define Fanconi’s syndrome may not be routinely available in LMICs.

Our study has several limitations aside from the retrospective observational design. We have reported eGFR estimated using the MDRD formula, although consistent results were obtained when in a sensitivity analysis using the CG formula. The small sample size precludes interpretation of the incidence and adjustments for risk factors associated with renal impairment have not been included in the analysis. We have used a single eGFR < 60 ml/min/1.73 m^2^ estimation in the analysis that can indicate incident CKD, AKI or creatinine blips or laboratory errors. Additionally we have not included urine analysis data, although glycosuria (normoglycemic glcosuria) was used as a trigger to investigate for partial Fanconi syndrome. Comparing two different populations at two different settings with differing standards of practice also limits external validity.

## Conclusions

In summary, renal dysfunction was more commonly associated with the use of TDF based regimens in Western India compared to PLHIV in the UK. As TDF based regimens are being scaled up in LMICs, early identification and management of various forms of TDF induced renal injury would need renal monitoring (especially those with background risk factors for renal disease) and physician training. Exploring alternative strategies like lowering TDF dose and use of alternative formulations like TDF alefenamide (TAF) also needs to be urgently studied in these settings.

## Competing interests

The authors declare that they have no competing interests.

## Authors’ contributions

SP, SB, MY, MJ conceived and designed the study, AM, VB, KJ, and DD abstracted data, CS was involved in statistical analysis. All authors read and approved the final manuscript.

## Pre-publication history

The pre-publication history for this paper can be accessed here:

http://www.biomedcentral.com/1471-2334/14/173/prepub

## References

[B1] De WR Cohen K Maartens G Systematic review of antiretroviral-associated lipodystrophy: lipoatrophy, but not central fat gain, is an antiretroviral adverse drug reaction PLoS One 2013 14 5 e63623 10.1371/journal.pone.0063623 23723990 PMC3665842

[B2] Antiretroviral therapy for HIV infection in adults and adolescents Recommendations for a public health approach: 2010 revision 2010 http://www.who.int/hiv/pub/arv/adult2010/en/index.html. **2013** 23741771

[B3] Gallant JE Staszewski S Pozniak AL DeJesus E Suleiman JM Miller MD Coakley DF Lu B Toole JJ Cheng AK 903 Study group Efficacy and safety of tenofovir DF vs stavudine in combination therapy in antiretroviral-naive patients: a 3-year randomized trial JAMA 2004 14 2 191 201 10.1001/jama.292.2.191 15249568

[B4] Agbaji OO Agaba PA Idoko JA Taiwo B Murphy R Kanki P Ekong E Temporal changes in renal glomerular function associated with the use of Tenofovir Disoproxil Fumarate in HIV-infected Nigerians West Afr J Med 2011 14 3 164 168 22120479

[B5] Gayet-Ageron A Ananworanich J Jupimai T Chetchotisakd P Prasithsirikul W Ubolyam S Le Braz M Ruxrungtham K Rooney JF Hirschel B Staccato study group No change in calculated creatinine clearance after tenofovir initiation among Thai patients J Antimicrob Chemother 2007 14 5 1034 1037 10.1093/jac/dkm064 17376791

[B6] Manosuthi W Prasithsirikul W Tantanathip P Chimsuntorn S Nilkamhang S Sungkanuparph S Renal impairment in HIV-1 infected patients receiving antiretroviral regimens including tenofovir in a resource-limited setting Southeast Asian J Trop Med Public Health 2011 14 3 643 650 21706942

[B7] Kiertiburanakul S Chaisiri K Kasettratat N Visuttimak P Bowonwatanuwong C Monitoring of Renal Function among HIV-Infected Patients Receiving Tenofovir in a Resource-Limited Setting J Int Assoc Physicians AIDS Care (Chic ) 2011 14 5 297 302 10.1177/1545109711406735 21606380

[B8] Cooper RD Wiebe N Smith N Keiser P Naicker S Tonelli M Systematic review and meta-analysis: renal safety of tenofovir disoproxil fumarate in HIV-infected patients Clin Infect Dis 2010 14 5 496 505 10.1086/655681 20673002

[B9] Ryom L Mocroft A Kirk O Ross M Reiss P Fux CA Morlat P Moranne O Smith C El-Sadr W Law M Lundgren JD Predictors of advanced chronic kidney disease and end-stage renal disease in HIV-positive persons AIDS 2014 14 2 187 199 10.1097/QAD.0000000000000042 24361680

[B10] Stohr W Reid A Walker AS Ssali F Munderi P Mambule I Kityo C Grosskurth H Gilks CF Gibb DM Hakim J DART Trial team Glomerular dysfunction and associated risk factors over 4-5 years following antiretroviral therapy initiation in Africa Antivir Ther 2011 14 7 1011 1020 10.3851/IMP1832 22024517

[B11] Bailey PK Tomson CR Kinra S Ebrahim S Radhakrishna KV Kuper H Nitsch D Ben-Schlomo Y Differences in estimation of creatinine generation between renal function estimating equations in an Indian population: cross-sectional data from the Hyderabad arm of the Indian migration study BMC Nephrol 2013 14 30 10.1186/1471-2369-14-30 23379609 PMC3599554

[B12] Hall AM Bass P Unwin RJ Drug-induced renal Fanconi syndrome. QJM 2014 10.1093/qjmed/hct258 24368854

[B13] Pujari S Dravid A Gupte N Joshix K Bele V Effectiveness and Safety of Generic Fixed-Dose Combination of Tenofovir/Emtricitabine/Efavirenz in HIV-1-Infected Patients in Western India J Int AIDS Soc 2008 14 8 196 10.1186/1758-2652-10-8-196 19825144 PMC2757396

[B14] Patel KK Patel AK Ranjan RR Patel AR Patel JK Tenofovir-associated renal dysfunction in clinical practice: An observational cohort from western India Indian J Sex Transm Dis 2010 14 1 30 34 10.4103/0253-7184.68998 21808434 PMC3140146

[B15] Calcagno A De Gonzalez RD Simiele M D’Avolio A Tettoni MC Salassa B Orofino G Bramato C Libanore V Motta I Bigliano P Orsucci E DiPerri G Bonora S Tenofovir plasma concentrations according to companion drugs: a cross-sectional study of HIV-positive patients with normal renal function Antimicrob Agents Chemother 2013 14 4 1840 1843 10.1128/AAC.02434-12 23380733 PMC3623307

[B16] Nishijima T Gatanaga H Komatsu H Tsukada K Shimbo T Aoki T Watanabe K Kinai E Honda H Tanuma J Yazaki H Honda M Teruya K Kikuchi Y Oka S Renal function declines more in tenofovir- than abacavir-based antiretroviral therapy in low-body weight treatment-naive patients with HIV infection PLoS One 2012 14 1 e29977 10.1371/journal.pone.0029977 22242194 PMC3252345

[B17] Goicoechea M Liu S Best B Sun S Jain S Kemper C Witt M Diamond C Haubrich R Louie S California Collaborative Treatment Group 578 Team Greater tenofovir-associated renal function decline with protease inhibitor-based versus nonnucleoside reverse-transcriptase inhibitor-based therapy J Infect Dis 2008 14 1 102 108 10.1086/524061 18171292

[B18] Kohler JJ Hosseini SH Green E Abuin A Ludaway T Russ R Santoianni R Lewis W Tenofovir renal proximal tubular toxicity is regulated by OAT1 and MRP4 transporters Lab Invest 2011 14 6 852 858 10.1038/labinvest.2011.48 21403643 PMC3103636

[B19] Young J Schafer J Fux CA Furrer H Bernasconi E Vernazza P Calmy A Cavassini M Weber R Battegay M Bucher HC Swiss HIV Cohort S Renal function in patients with HIV starting therapy with tenofovir and either efavirenz, lopinavir or atazanavir AIDS 2012 14 5 567 575 10.1097/QAD.0b013e32834f337c 22398568

[B20] Hall AM Hendry BM Nitsch D Connolly JO Tenofovir-associated kidney toxicity in HIV-infected patients: a review of the evidence Am J Kidney Dis 2011 14 5 773 780 10.1053/j.ajkd.2011.01.022 21435764

